# Vitexin attenuates lipopolysaccharide-induced acute lung injury by controlling the Nrf2 pathway

**DOI:** 10.1371/journal.pone.0196405

**Published:** 2018-04-25

**Authors:** Ying Lu, Ting Yu, Jingyao Liu, Lina Gu

**Affiliations:** 1 Intensive Care Unit, the First Hospital of Jilin University, Changchun, China; 2 Department of Nutrition, the Second Hospital of Jilin University, Changchun, China; 3 Departmen of Neurology, the First Hospital of Jilin University, Changchun, China; National Institutes of Health, UNITED STATES

## Abstract

**Background:**

A major feature of acute lung injury (ALI) is excessive inflammation in the lung. Vitexin is an active component from medicinal plants which has antioxidant and anti-inflammatory activities. Oxidative stress and inflammation play important roles in the pathophysiological processes in ALI. In the current study, we investigate the effect and potential mechanisms of Vitexin on lipopolysaccharide (LPS)-induced ALI.

**Methods:**

ALI was induced by LPS intratracheal instillation in C57BL/6 wild-type mice and Nrf2 gene knocked down (Nrf2-/-) mice. One hour before LPS challenge, Vitexin or vehicle intraperitoneal injection was performed. Bronchoalveolar lavage fluid and lung tissues were examined for lung inflammation and injury at 24 h after LPS challenge.

**Results:**

Our animal study’s results showed that LPS-induced recruitment of neutrophils and elevation of proinflammatory cytokine levels were attenuated by Vitexin treatment. Vitexin decreased lung edema and alveolar protein content. Moreover, Vitexin activated nuclear factor erythroid-2-related factor 2 (Nrf2), and increased the activity of its target gene heme oxygenase (HO)-1. The LPS-induced reactive oxygen species were inhibited by Vitexin. In addition, the activation of the nucleotide-binding domain and leucine-rich repeat PYD-containing protein 3 (NLRP3) inflammasome was suppressed by Vitexin. However, these effects of Vitexin were abolished in the Nrf2-/- mice. Our cell studies showed that Vitexin enhanced the expression of Nrf2 and HO-1 activity. Moreover, reactive oxygen species (ROS) and IL-1β productions were reduced in Vitexin-treated cells. However, knockdown of Nrf2 by siRNA in RAW cells reversed the benefit of Vitexin.

**Conclusions:**

Vitexin suppresses LPS-induced ALI by controlling Nrf2 pathway.

## Introduction

Acute lung injury (ALI)/acute respiratory distress syndrome (ARDS) is characterized by diffuse lung inflammation[1, 2]. Controlling inflammation is a promising strategy for ALI/ ARDS[1–4].

The nucleotide binding domain and leucine-rich repeat pyrin domain containing 3 (NLRP3) inflammasome has been demonstrated to associate with ALI[5–7]. NLRP3 inflammasome plays an important effect in the regulation of interleukin (IL)-1β[8]. IL-1β is one of the most important inflammatory mediators in the development of ALI[9]. Reactive oxygen species (ROS) which were generated in ALI have been identified as an important activator of NLRP3 inflammasome[10]. Nuclear factor erythroid-2-related factor 2 (Nrf2) is a redox-sensitive transcription factor. Nrf2 has been shown to regulate expression of antioxidants such as heme oxygenase (HO)-1 which is critical in protecting the lung against oxidative stress[11]. Various investigators have demonstrated the importance of Nrf2 activation and up-regulation of HO-1 in ALI[11].

Vitexin is an active component from medicinal plants such as the leaf of hawthorn which is a widely used conventional Chinese medicine[12]. Modern pharmacological studies show that Vitexin exerts a variety of pharmacological activities, including antioxidant and anti-inflammatory functions[13–18]. Vitexin reduced hypoxia-ischemia neonatal brain damage[19], acute myocardial ischemia/reperfusion injury[18], lipopolysaccharide (LPS)-induced islet cell injury[14]. The antioxidant and anti-inflammatory features of Vitexin may contribute to the reduction of ALI. The objective of the present study was to examine the effects of Vitexin in ALI. We hypothesized that Vitexin prevents ALI via controlling Nrf2 pathway.

## Materials and methods

### Animal experimental protocol

All animal experiments of the present study were performed in accordance with the National Institutes of Health guidelines for the use of experimental animals. The present project was approved by the Institutional Animal Care and Use Committee of Jilin University. To minimize the suffering of animals, various intervals were performed under anesthesia.

Male wild-type (WT) C57BL/6 mice (18–20 g, the laboratory animal center of Jilin University) and Nrf2 gene knock out (Nrf2-/-) mice (18–20 g, the Jackson Laboratory, Bar Harbor, ME, USA) were anesthetized (n = 10 each group). The ALI model was induced by intratracheal administration of LPS (Escherichia coli 0111:B4; Sigma-Aldrich, St. Louis, MO, USA) at a dose of 10 mg/mL which was dissolved in 50 μL sterile phosphate-buffered saline (PBS) as described previously[20]. For control group, 50 μL PBS was administrated. One hour before LPS or PBS challenge, Vitexin (10 mg/kg, Haoxuan Biotechnology Co. Ltd., Xi’an, China) or vehicle (20 μL dimethylsulphoxide in a total of 200 μL saline) intraperitoneal injection (i.p.) was performed. Twenty-four hours after LPS or PBS challenge, mice were sacrificed after anesthesia by pentobarbitone (50 mg/kg i.p.).

### Bronchoalveolar lavage fluid (BALF)

We collected BALF by lavage the lung for three times with PBS (pH 7.2, 500 μL each time). The fluid recovery rate was more than 90%. Lavage samples from mice were kept on ice. BALF was centrifuged at 700× g for 5 min at 4 °C. The BALF supernatant was collected and stored at −80 °C.

### Measurement of cytokine concentration and protein content in BALF

Tumor necrosis factor (TNF)-α, IL-1β, and IL-6 in BALF were measured by an enzyme-linked immunosorbent assay (ELISA) (R&D Systems, Minneapolis, MN, USA) according to the manufacturers’ manual. The measurement of total protein content in BALF was performed by using Pierce BCA protein assay kit (Thermo Fisher Scientific, Asheville, NC, USA) as previously described[21].

### Pulmonary wet to dry (W/D) weight ratio

The left upper lung lobe was harvested for lung W/D weight ratio. The lung was weighed, placed in an oven at 80°C for 48 h, and then dried and re-weighed. The W/D ratio was then calculated[22].

### Histopathological analysis

The lung tissues were fixed with 10% buffered formalin, embedded in paraffin, and then, sectioned into 5 μm slices. The lung tissue slices were stained with haematoxylin-eosin, and analyzed by light microscopy. A scoring system to grade the degree of lung injury was employed in the present study. Briefly, the lung tissue sections were graded on a scale of 0 to 4 (0, absent and appears normal; 1, light; 2, moderate; 3, strong; 4, intense) for congestion, edema, infiltration of inflammatory cells, and hemorrhaging[23].

### Measurement of maleic dialdehyde (MDA) production

The pulmonary homogenate (0.1 ml) was mixed with 0.2 ml of 8.1% sodium dodecyl sulfate, 1.5 ml of 20% acetic acid, and 1.5 ml of 0.8% aqueous solution of thio-barbituric acid. The mixture pH was adjusted to 3.5, and the final volume was made up to 4.0 ml with distilled water; 5.0 ml of the mixture of n-butanol and pyridine (15:1, v/v) was then added. The mixture was shaken vigorously. After centrifugation at 4,000 rpm for 10 min, the absorbance of the organic layer was measured at 532 nm. MDA was expressed as nmol/mg protein[24].

### Cell viability

RAW 264.7 cells (the China Cell Line Bank, Beijing, China) were cultured in Dulbecco’s modified Eagle’s medium supplemented with 10% fetal bovine serum, 100 U/ml of penicillin, and 100 U/ml of streptomycin in a 5% CO_2_ humidified atmosphere at 37°C prior to experiments.

Cell viability was determined using the (4, 5-dimethylthiazol-2-yl)-2,5-diphenyltetrazoliumbromide (MTT) assay. The cells were treated with various concentrations of Vitexin for 1 hour and then exposed to LPS (10 μg/mL) for 12 hours. Subsequently, 100 μL of MTT solution (0.5 mg/ml) was added and further incubated for 4 h. The result was measured using a microplate reader (Biotek, Winooski, VT, USA) at an absorbance of 570 nm.

### Small interfering RNA (siRNA) transfection

RAW 264.7 cells were transfected with Nrf2 siRNA and Non-Correlated siRNA according to the manufacturers’ manual (Santa Cruz Biotechnology, Santa Cruz, CA, USA). In brief, cells (2×10^5^ cells/well) were seeded in 6-well plates. The transfection of siRNAs was carried out by using the siRNA transfection reagent Lipofectamine^™^ 2000 following the manufacturer’s protocol. After 6 h, cells were split into 6-well plates to perform further analysis. We detected the effects of siRNA on Nrf2 expression by western blot analysis. The transfected cells were stimulated with LPS for 12 h in the presence and absence of Vitexin.

### ROS measurement

The RAW 264.7 cells were incubated with DCFH-DA (20 μM) for 30 min. The fluorescence intensity was scanned by a fluorometer (Molecular Devices Gemini XS, Sunnyvale, CA, USA) at excitation and emission wavelength of 485 nm and 538 nm, respectively.

### HO-1 activity assay

HO-1 activity was detected by measuring the amount of bilirubin. Briefly, the lung tissue homogenate (600 μL) was mixed with 0.8 mmol/L nicotinamide adenine dinucleotide phosphate, 1 mmol/L glucose-6-phosphate, 0.2 U glucose 6-phosphate dehydrogenase, and 2.5 mmol/L protohemin. The reaction was incubated for 1 h at 37°C in a water bath with shaking in the dark. The reaction was terminated by adding chloroform. The amounts of generated bilirubin were determined by absorbance at 464 and 530 nm.

### Nrf2 activity analysis

The nuclear extractions from lung tissues were used for measuring Nrf2 binding activity to immobilized antioxidant response elements (ARE) using a TransAM^™^ Nrf2 kit according to the manufacturer’s instructions (Active Motif, Carlsbad, CA, USA).

### Western blotting analysis

Protein concentration was determined using a Bio-Rad protein assay (Bio-Rad Laboratories, Hercules, CA). Equal concentrations of proteins were mixed with SDS sample buffer and denatured at 95 °C for 5 min. The samples were resolved with 8% SDS–page gels which were then transferred onto nitrocellulose membranes. The membranes were blocked with 5% fat-free milk in 0.1% Tris-buffered saline with Tween (TTBS) for 2 hours and then probed with primary antibodies: anti-Nrf2 antibody (1:500 dilution; Santa Cruz Biotechnology, Santa Cruz, CA), anti-NLRP3 antibody (Cell Signaling Technology, Beverley, CA) at 4 °C overnight. After being washed for three times with TTBS, the membranes were incubated with secondary antibodies (Santa Cruz Biotechnology) in TTBS at room temperature for 2 hours. Membranes were washed again with TTBS three times and then visualized on X-ray films using a chemo-luminescence detection system (ECL, GE Healthcare). β-actin (Santa Cruz Biotechnology) and TATA box binding protein (TBP) (Abcam, Cambridge, UK) were used as protein loading control for cytoplasmic and nuclear proteins, respectively. The relative band intensities were measured by image analysis software Gel-Pro Analyser.

### Statistical analysis

All data are presented as means ± SEM. All data were analyzed by using the SPSS 17.0 software (Chicago, IL, USA). The two-tailed Student’s t-test for comparison between two groups. The one-way analysis of variance (ANOVA) followed by Bonferroni’s post hoc test for multiple comparisons was used to compare more than two groups. The Student-Newman-Keuls method was used for statistical evaluation of the histopathological analysis. No statistical method was used to estimate sample size, but it was consistent with previous publications. Three replicates per condition were performed for *in vitro* studies. A value of *P* < 0.05 was taken to indicate statistical significance.

## Results

### Effect of Vitexin on pulmonary histological alteration in LPS-treated mice

**Fig 1:**
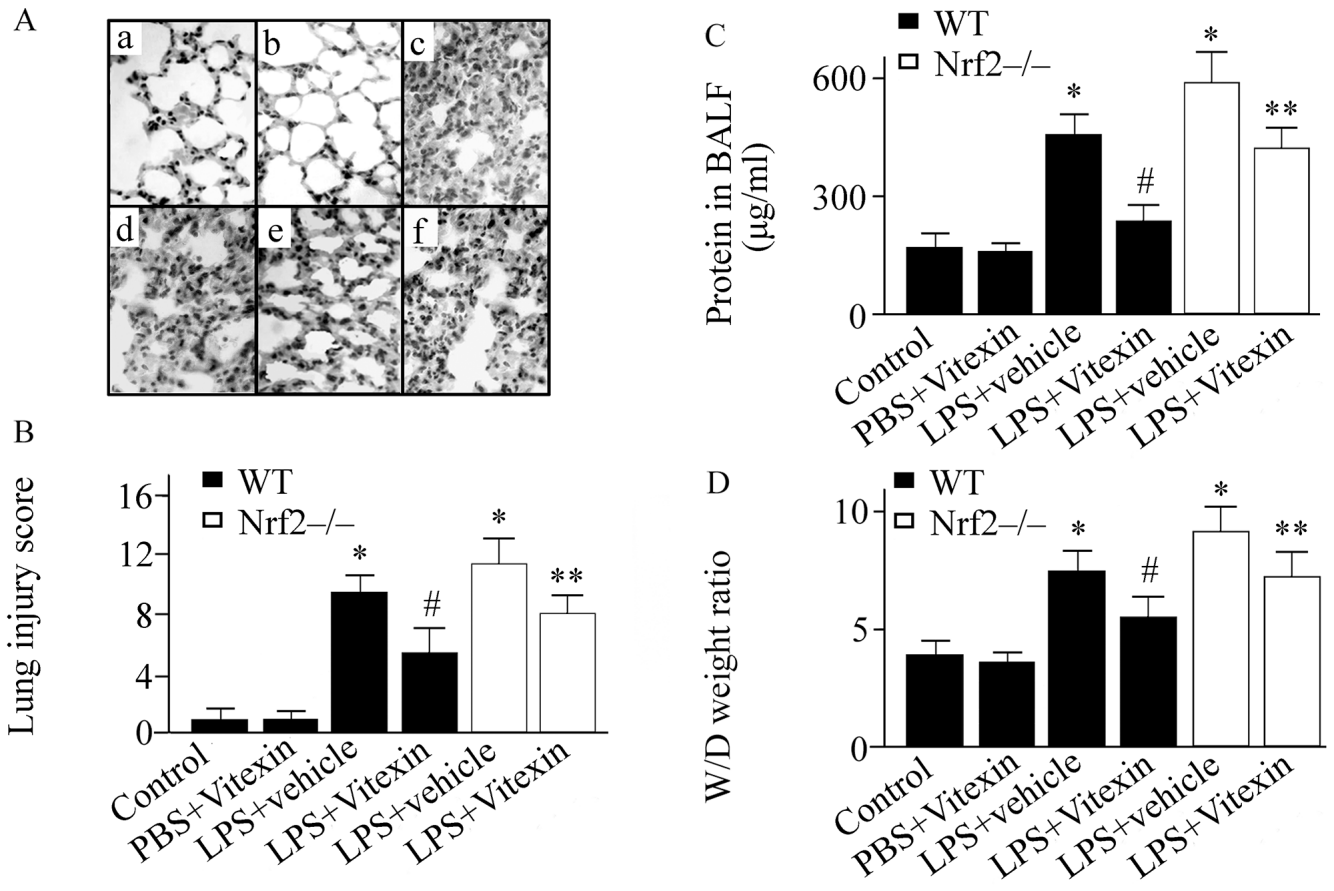
Effects of Vitexin on pulmonary histopathological analysis, lung injury score, lung permeability, and lung water content in lipopolysaccharide (LPS)-treated mice. Representative haematoxylin-eosin staining images of pulmonary section (A): a, control group (wild type (WT) mice treated with sterile phosphate-buffered saline (PBS)+vehicle); b, WT mice treated with PBS+Vitexin; c, nuclear factor erythroid-2-related factor 2 (Nrf2) gene knockout (Nrf2-/-) mice treated with LPS+vehicle; d, WT mice treated with LPS+vehicle; e, WT mice treated with LPS+Vitexin; f, Nrf2-/- mice treated with LPS+Vitexin. All photographs were taken at 100×magnification. Lung injury score (B). Protein concentrations in bronchoalveolar lavage fluid (BALF) (C). Pulmonary wet to dry (W/D) weight ratio (D). Data was expressed as means ± SEM (n = 6–10 per group). * *p* < 0.05, versus control group; ^#^*p* < 0.05, versus LPS+vehicle group; ** *p* < 0.05, versus LPS+Vitexin treated WT mice.

There was no histological alteration in the control group (Fig 1A and 1B). However, the pulmonary inflammation and injury were observed at 24 h after LPS challenge in WT mice (Fig 1A and 1B). These LPS-induced histological changes were ameliorated by Vitexin treatment (Fig 1A and 1B). However, the benefit of Vitexin on pulmonary histological alteration was abolished in Nrf2-/- mice (Fig 1A and 1B).

### Effect of Vitexin on lung water content and permeability in LPS-treated mice

LPS challenge induced a significant increase in BALF protein concentrations and pulmonary wet to dry ratio in WT mice (Fig 1C and 1D). Vitexin treatment inhibited the LPS-induced elevation of protein concentrations in BALF and pulmonary wet to dry ratio by 48% (232.88+43.43 (LPS+Vitexin) vs. 451.66+38.85 (LPS+vehicle)) and 26% (5.48+0.83 (LPS+Vitexin) vs. 7.51+0.90 (LPS+vehicle)), respectively (Fig 1C and 1D). However, the benefit of Vitexin was markedly inhibited in the Nrf2-/- mice (Fig 1C and 1D).

### Effect of Vitexin on cytokine concentration in LPS-treated mice

**Fig 2:**
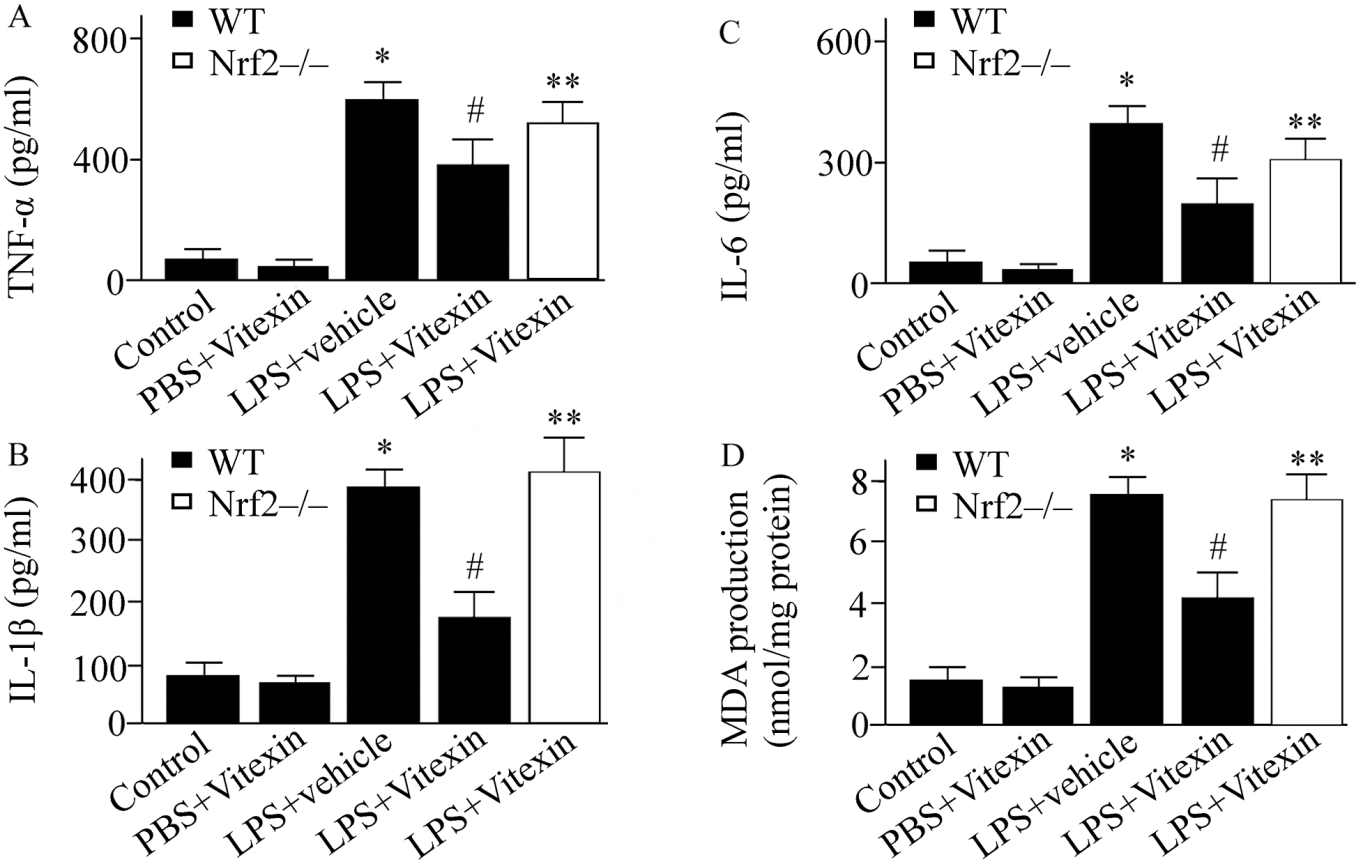
Effects of Vitexin on tumor necrosis factor (TNF)-α (A), interleukin (IL)-1β (B), IL-6 (C), and maleic dialdehyde (MDA) production (D) in lipopolysaccharide (LPS)-treated mice. Data was expressed as means ± SEM (n = 6–10 per group). * *p* < 0.05, versus control group (wild type (WT) mice treated with PBS+vehicle); ^#^*p* < 0.05, versus LPS+vehicle group; ** *p* < 0.05, versus LPS+Vitexin treated WT mice. Nrf2-/-, nuclear factor erythroid-2-related factor 2 gene knockout mice.

24 hours after intratracheal injection of LPS, TNF-α, IL-1β, and IL-6 levels in BALF were determined using ELISA. The concentrations of TNF-α, IL-1β, and IL-6 in the vehicle treated group were higher than Vitexin treated group (Fig 2A, 2B and 2C). However, the LPS-induced the elevation of TNF-α, IL-1β, and IL-6 in Vitexin treated Nrf2-/- mice was higher than Vitexin treated WT mice (Fig 2A, 2B and 2C).

### Effect of Vitexin on Nrf2/HO-1 pathway and ROS production in LPS-treated mice

**Fig 3:**
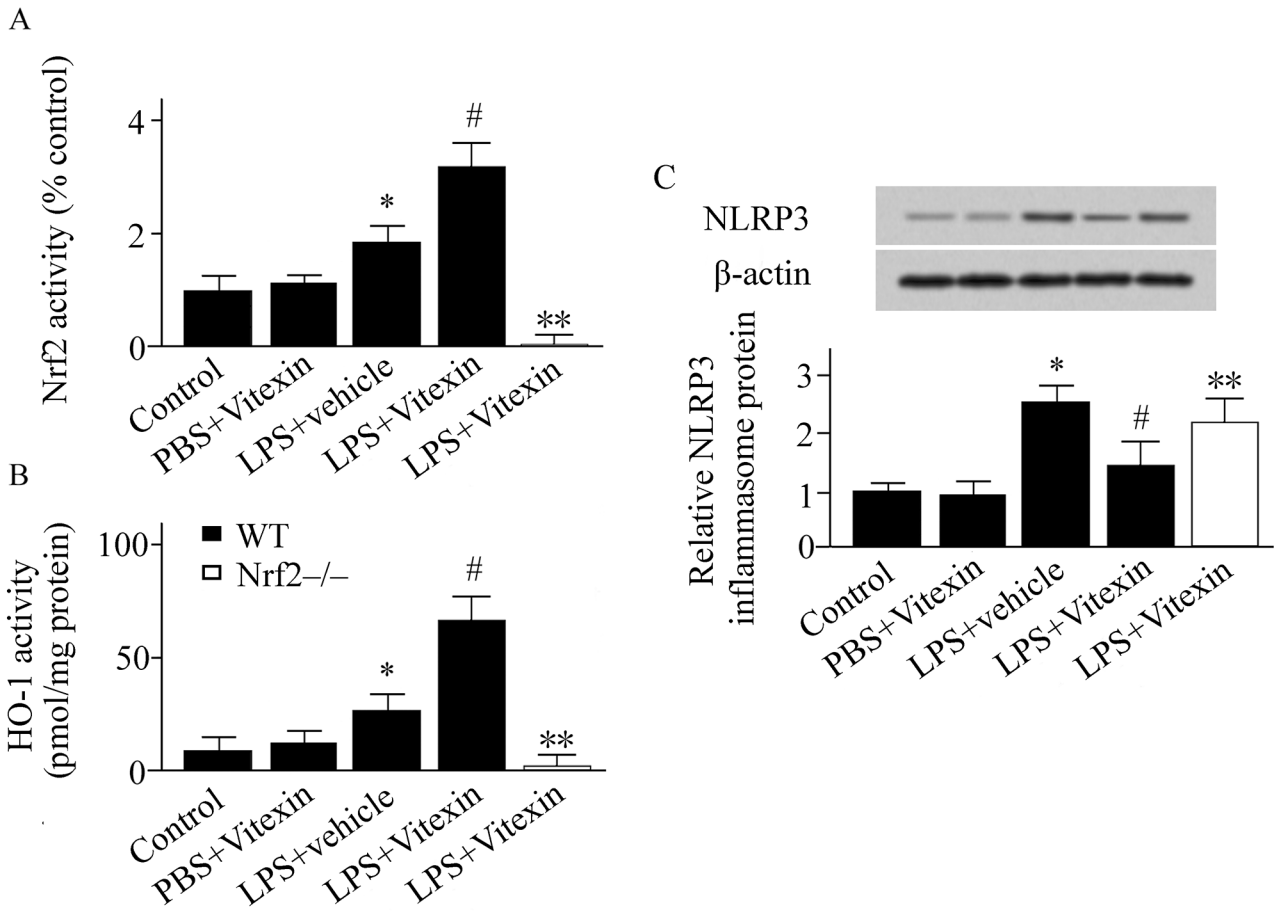
Effects of Vitexin on nuclear factor erythroid-2-related factor 2 (Nrf2) activity (A), heme oxygenase (HO)-1 activity (B), and the nucleotide-binding domain and leucine-rich repeat PYD-containing protein 3 (NLRP3) inflammasome (C) in lipopolysaccharide (LPS)-treated mice. Data was expressed as means ± SEM (n = 6–10 per group). * *p* < 0.05, versus control group (wild type (WT) mice treated with PBS+vehicle); ^#^*p* < 0.05, versus LPS+vehicle group; ** *p* < 0.05, versus LPS+Vitexin treated WT mice. Nrf2-/-, Nrf2 gene knockout mice.

ROS levels in lung tissue samples were expressed in MDA. LPS challenge increased ROS generation by 5.2-fold compared with control (Fig 2D). Vitexin treatment reduced the LPS-induced ROS levels by 44% compared with vehicle-treated mice (Fig 2D). However, the benefit of Vitexin on ROS production was abolished in Nrf2-/- mice (Fig 2D). Nrf2 and HO-1 activity were determined 24 h after LPS challenge. Compared with vehicle-treated group, Vitexin treatment markedly upregulated the activity of Nrf2 and HO-1 (Fig 3A and 3B).

### Effect of Vitexin on NLRP3 inflammasome in LPS-treated mice

Western blotting analysis revealed that the NLRP3 inflammasome was upregulated in the mice with ALI compared with that in the control (Fig 3C). Vitexin treatment inhibited the NLRP3 inflammasome compared with vehicle-treated animals observed at 24 h after LPS challenge (Fig 3C). The inhibiting effect of Vitexin on the NLRP3 inflammasome was markedly dampened in Nrf2-/- mice (Fig 3C).

### Effect of Vitexin on LPS-activated RAW cells

**Fig 4:**
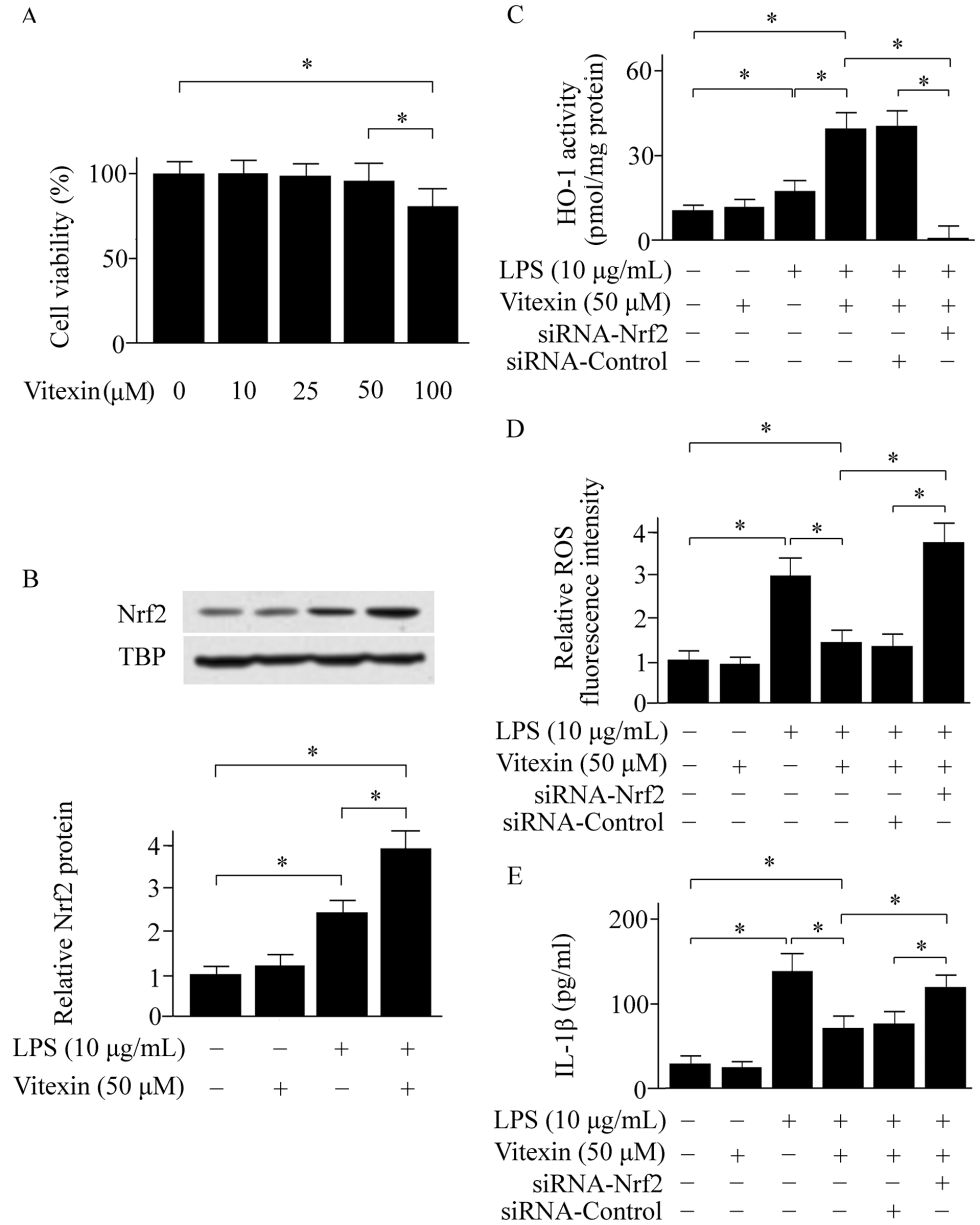
Effects of Vitexin on cell viability (A), the expressions of nuclear factor erythroid-2-related factor 2 (Nrf2) (B), heme oxygenase (HO)-1 activity (C), reactive oxygen species (ROS) levels (D), and interleukin (IL)-1β levels (E) in lipopolysaccharide (LPS)-activated RAW cells. TBP, TATA box binding protein. Data was expressed as means ± SEM of three independent experiments. * *p* < 0.05.

The MTT assay showed that Vitexin did not exhibit any toxicity to RAW 264.7 cells at concentrations ranging from 10 to 50 μM (Fig 4A). Our cell study showed that Vitexin elevated Nrf2 expression in RAW 264.7 cells (Fig 4B). Meanwhile, the HO-1 activity was enhanced in Vitexin-treated group (Fig 4C). The ROS levels in RAW 264.7 cells were determined using DCFH-DA. Vitexin treatment inhibited ROS and IL-1β production (Fig 4D and 4E). However, these effects of Vitexin were abolished when the Nrf2 gene was knocked down (Fig 4C, 4D and 4E).

## Discussion

In the current study, pulmonary edema and inflammation was observed in vehicle-treated WT mice at 24 hours after LPS challenge. These changes were dampened by Vitexin treatment. Moreover, Vitexin treatment elevated Nrf2 expression, and inhibited NLRP3 inflammasome activation. However, the benefit of Vitexin on elevation of HO-1 and reduction of ROS as well as cytokine production were abolished in the Nrf2-/- mice. Our results suggest that the benefit of Vitexin on LPS-induced ALI is mediated through controlling Nrf2 pathway.

In this study, our data suggest that Vitexin may have antioxidative effects via the upregulation of Nrf2. Oxidative stress plays a vital role in ALI[25]. Excessive ROS during the pathogenesis of ALI is a hallmark feature of oxidative stress[25]. ROS causes cell swelling and cell membrane breakdown[26]. ROS accumulation aggravates inflammatory responses via promoting the expression of proinflammatory cytokines and infiltration of inflammatory cells[27]. The important role of Nrf2 in ALI has been demonstrated by previous studies[28]. Nrf2 regulates the expression of antioxidant enzymes which counteracts ROS generation[27]. HO-1 is an important target gene of Nrf2 which plays a crucial role in host defense against inflammation and oxidative stress[29–31]. Evidences have shown that Vitexin has antioxidant features[18, 19]. However, its potential mechanism is unclear. In the present study, the Nrf2/HO-1 pathway was activated by Vitexin treatment in animal study. Meanwhile, the LPS-induced ROS production was inhibited by Vitexin treatment. However, the benefit of Vitexin was abolished in Nrf2-/- mice. Our *in vitro* data are consistent with animal studies. Knockdown of Nrf2 by siRNA in RAW cells reversed the benefit of Vitexin. Our results showed that the benefit of Vitexin is associated with regulating the Nrf2 pathway.

The NLRP3 inflammasome is a pivotal signaling platform that is activated by a variety of signals, such as ROS[32]. Activation of NLRP3 inflammasome has been shown to be involved in ALI[7, 33–35]. Evidence has shown that activation of NLRP3 inflammasome is associated with releasing of cytokines[36]. Recent study suggests that NLRP3 inflammasome causes maturation of the proinflammatory cytokines IL-1β[37]. IL-1β is an important proinflammatory cytokine in ALI. IL-1β causes alveolar epithelial and vascular endothelial permeability[9, 38]. In the current study, the LPS-induced activation of NLRP3 inflammasome was inhibited in Vitexin-treated mice. The IL-1β levels were reduced in Vitexin-treated groups. It appears that the beneficial effect of Vitexin on LPS-induced ALI is associated with inhibition of NLRP3 inflammasome. The inhibiting role of Vitexin on NLRP3 inflammasome may via Nrf2-mediated reduction of ROS production.

There are limitations in the present study. First, our results showed that Vitexin upregulated Nrf2 expression in LPS-induced ALI. However, the underlying signaling pathway involved in the effect of Vitexin on Nrf2 warrants further investigation. Second, only one time point was investigated in the present study. The effect of Vitexin on ALI beyond than 24 hours is unclear. Last, other mechanisms responsible for the effect of Vitexin on ALI were not elucidated.

## Conclusions

Vitexin suppresses LPS-induced ALI by controlling Nrf2 pathway. Vitexin may represent a promising therapeutic strategy for ameliorating development of ALI.

## Supporting information

S1 Fig The original blot of Fig 3C. (TIF)

S2 Fig The original blot of Fig 4B. (TIF)

## References

[1] Leaver SK, Evans TW. Acute respiratory distress syndrome. Bmj. 2007;335(7616):389–94. doi: 10.1136/bmj.39293.624699.AD . 17717368 10.1136/bmj.39293.624699.AD PMC1952500

[2] Sweeney RM, McAuley DF. Acute respiratory distress syndrome. Lancet. 2016;388(10058):2416–30. doi: 10.1016/S0140-6736(16)00578-X . 27133972 10.1016/S0140-6736(16)00578-X PMC7138018

[3] Tao W, Li PS, Yang LQ, Ma YB. Effects of a Soluble Epoxide Hydrolase Inhibitor on Lipopolysaccharide-Induced Acute Lung Injury in Mice. PLoS One. 2016;11(8):e0160359 doi: 10.1371/journal.pone.0160359 . 27490848 10.1371/journal.pone.0160359 PMC4973880

[4] Tao W, Li PS, Xu G, Luo Y, Shu YS, Tao YZ, et al Soluble Epoxide Hydrolase Plays a Vital role In Angiotensin II-Induced Lung Injury in Mice. Shock (Augusta, Ga). 2017 Epub 2017/12/06. doi: 10.1097/shk.0000000000001067 . 29206762 10.1097/SHK.0000000000001067

[5] Xiang M, Shi X, Li Y, Xu J, Yin L, Xiao G, et al Hemorrhagic shock activation of NLRP3 inflammasome in lung endothelial cells. Journal of immunology (Baltimore, Md: 1950). 2011;187(9):4809–17. Epub 2011/09/24. doi: 10.4049/jimmunol.1102093 21940680 10.4049/jimmunol.1102093 PMC3197874

[6] Han S, Cai W, Yang X, Jia Y, Zheng Z, Wang H, et al ROS-Mediated NLRP3 Inflammasome Activity Is Essential for Burn-Induced Acute Lung Injury. Mediators of inflammation. 2015;2015:720457 Epub 2015/11/18. doi: 10.1155/2015/720457 26576075 10.1155/2015/720457 PMC4630408

[7] Jones HD, Crother TR, Gonzalez-Villalobos RA, Jupelli M, Chen S, Dagvadorj J, et al The NLRP3 inflammasome is required for the development of hypoxemia in LPS/mechanical ventilation acute lung injury. American journal of respiratory cell and molecular biology. 2014;50(2):270–80. Epub 2013/09/07. doi: 10.1165/rcmb.2013-0087OC 24007300 10.1165/rcmb.2013-0087OC PMC3930947

[8] Martinon F, Burns K, Tschopp J. The inflammasome: a molecular platform triggering activation of inflammatory caspases and processing of proIL-beta. Mol Cell. 2002;10(2):417–26. . 12191486 10.1016/s1097-2765(02)00599-3

[9] Ganter MT, Roux J, Miyazawa B, Howard M, Frank JA, Su G, et al Interleukin-1beta causes acute lung injury via alphavbeta5 and alphavbeta6 integrin-dependent mechanisms. Circ Res. 2008;102(7):804–12. doi: 10.1161/CIRCRESAHA.107.161067 . 18276918 10.1161/CIRCRESAHA.107.161067 PMC2739091

[10] Strowig T, Henao-Mejia J, Elinav E, Flavell R. Inflammasomes in health and disease. Nature. 2012;481(7381):278–86. Epub 2012/01/20. doi: 10.1038/nature10759 . 22258606 10.1038/nature10759

[11] Ma Q. Role of nrf2 in oxidative stress and toxicity. Annual review of pharmacology and toxicology. 2013;53:401–26. Epub 2013/01/09. doi: 10.1146/annurev-pharmtox-011112-140320 23294312 10.1146/annurev-pharmtox-011112-140320 PMC4680839

[12] Wu J, Peng W, Qin R, Zhou H. Crataegus pinnatifida: chemical constituents, pharmacology, and potential applications. Molecules (Basel, Switzerland). 2014;19(2):1685–712. Epub 2014/02/04. doi: 10.3390/molecules19021685 . 24487567 10.3390/molecules19021685 PMC6271784

[13] Venturini CL, Macho A, Arunachalam K, de Almeida DAT, Rosa SIG, Pavan E, et al Vitexin inhibits inflammation in murine ovalbumin-induced allergic asthma. Biomedicine & pharmacotherapy = Biomedecine & pharmacotherapie. 2017;97:143–51. Epub 2017/11/02. doi: 10.1016/j.biopha.2017.10.073 . 29091859 10.1016/j.biopha.2017.10.073

[14] Wang F, Yin J, Ma Y, Jiang H, Li Y. Vitexin alleviates lipopolysaccharideinduced islet cell injury by inhibiting HMGB1 release. Molecular medicine reports. 2017;15(3):1079–86. Epub 2017/01/19. doi: 10.3892/mmr.2017.6114 28098903 10.3892/mmr.2017.6114 PMC5367348

[15] Zhang S, Guo C, Chen Z, Zhang P, Li J, Li Y. Vitexin alleviates ox-LDL-mediated endothelial injury by inducing autophagy via AMPK signaling activation. Molecular immunology. 2017;85:214–21. Epub 2017/03/14. doi: 10.1016/j.molimm.2017.02.020 . 28288411 10.1016/j.molimm.2017.02.020

[16] Prabhakar MC, Bano H, Kumar I, Shamsi MA, Khan MS. Pharmacological investigations on vitexin. Planta medica. 1981;43(4):396–403. Epub 1981/12/01. . 7330108 10.1055/s-2007-971532

[17] Kim JH, Lee BC, Sim GS, Lee DH, Lee KE, Yun YP, et al The isolation and antioxidative effects of vitexin from Acer palmatum. Archives of pharmacal research. 2005;28(2):195–202. Epub 2005/03/26. . 15789751 10.1007/BF02977715

[18] Che X, Wang X, Zhang J, Peng C, Zhen Y, Shao X, et al Vitexin exerts cardioprotective effect on chronic myocardial ischemia/reperfusion injury in rats via inhibiting myocardial apoptosis and lipid peroxidation. American journal of translational research. 2016;8(8):3319–28. Epub 2016/09/21. 27648122 PMC5009384

[19] Min JW, Hu JJ, He M, Sanchez RM, Huang WX, Liu YQ, et al Vitexin reduces hypoxia-ischemia neonatal brain injury by the inhibition of HIF-1alpha in a rat pup model. Neuropharmacology. 2015;99:38–50. Epub 2015/07/19. doi: 10.1016/j.neuropharm.2015.07.007 . 26187393 10.1016/j.neuropharm.2015.07.007

[20] Lin WC, Lin CF, Chen CL, Chen CW, Lin YS. Inhibition of neutrophil apoptosis via sphingolipid signaling in acute lung injury. The Journal of pharmacology and experimental therapeutics. 2011;339(1):45–53. Epub 2011/07/05. doi: 10.1124/jpet.111.181560 . 21724966 10.1124/jpet.111.181560

[21] Peng X, Hassoun PM, Sammani S, McVerry BJ, Burne MJ, Rabb H, et al Protective effects of sphingosine 1-phosphate in murine endotoxin-induced inflammatory lung injury. American journal of respiratory and critical care medicine. 2004;169(11):1245–51. Epub 2004/03/17. doi: 10.1164/rccm.200309-1258OC . 15020292 10.1164/rccm.200309-1258OC

[22] Tao W, Shu YS, Miao QB, Zhu YB. Attenuation of hyperoxia-induced lung injury in rats by adrenomedullin. Inflammation. 2012;35(1):150–7. doi: 10.1007/s10753-011-9300-1 . 21302135 10.1007/s10753-011-9300-1

[23] Murakami K, McGuire R, Cox RA, Jodoin JM, Bjertnaes LJ, Katahira J, et al Heparin nebulization attenuates acute lung injury in sepsis following smoke inhalation in sheep. Shock (Augusta, Ga). 2002;18(3):236–41. Epub 2002/10/02. . 12353924 10.1097/00024382-200209000-00006

[24] Kurutas EB, Cetinkaya A, Bulbuloglu E, Kantarceken B. Effects of antioxidant therapy on leukocyte myeloperoxidase and Cu/Zn-superoxide dismutase and plasma malondialdehyde levels in experimental colitis. Mediators of inflammation. 2005;2005(6):390–4. Epub 2006/02/21. doi: 10.1155/MI.2005.390 16489261 10.1155/MI.2005.390 PMC1533903

[25] Cochrane CG, Spragg R, Revak SD. Pathogenesis of the adult respiratory distress syndrome. Evidence of oxidant activity in bronchoalveolar lavage fluid. J Clin Invest. 1983;71(3):754–61. doi: 10.1172/JCI110823 . 6600748 10.1172/JCI110823 PMC436926

[26] Hess ML, Okabe E, Kontos HA. Proton and free oxygen radical interaction with the calcium transport system of cardiac sarcoplasmic reticulum. Journal of molecular and cellular cardiology. 1981;13(8):767–72. Epub 1981/08/01. . 6267305 10.1016/0022-2828(81)90258-3

[27] Zhai Z, Gomez-Mejiba SE, Gimenez MS, Deterding LJ, Tomer KB, Mason RP, et al Free radical-operated proteotoxic stress in macrophages primed with lipopolysaccharide. Free Radic Biol Med. 2012;53(1):172–81. Epub 2012/05/15. doi: 10.1016/j.freeradbiomed.2012.04.023 22580125 10.1016/j.freeradbiomed.2012.04.023 PMC4078023

[28] Zhao H, Eguchi S, Alam A, Ma D. The role of nuclear factor-erythroid 2 related factor 2 (Nrf-2) in the protection against lung injury. Am J Physiol Lung Cell Mol Physiol. 2017;312(2):L155–l62. Epub 2016/11/20. doi: 10.1152/ajplung.00449.2016 . 27864288 10.1152/ajplung.00449.2016

[29] Nikam A, Ollivier A, Rivard M, Wilson JL, Mebarki K, Martens T, et al Diverse Nrf2 Activators Coordinated to Cobalt Carbonyls Induce Heme Oxygenase-1 and Release Carbon Monoxide in Vitro and in Vivo. Journal of medicinal chemistry. 2016;59(2):756–62. Epub 2016/01/06. doi: 10.1021/acs.jmedchem.5b01509 . 26730678 10.1021/acs.jmedchem.5b01509

[30] Sawle P, Moulton BE, Jarzykowska M, Green CJ, Foresti R, Fairlamb IJ, et al Structure-activity relationships of methoxychalcones as inducers of heme oxygenase-1. Chemical research in toxicology. 2008;21(7):1484–94. Epub 2008/06/13. doi: 10.1021/tx800115g . 18547064 10.1021/tx800115g

[31] Clark JE, Foresti R, Green CJ, Motterlini R. Dynamics of haem oxygenase-1 expression and bilirubin production in cellular protection against oxidative stress. The Biochemical journal. 2000;348 Pt 3:615–9. Epub 2000/06/07. 10839994 PMC1221105

[32] Latz E, Xiao TS, Stutz A. Activation and regulation of the inflammasomes. Nature reviews Immunology. 2013;13(6):397–411. Epub 2013/05/25. doi: 10.1038/nri3452 23702978 10.1038/nri3452 PMC3807999

[33] Galam L, Rajan A, Failla A, Soundararajan R, Lockey RF, Kolliputi N. Deletion of P2X7 attenuates hyperoxia-induced acute lung injury via inflammasome suppression. Am J Physiol Lung Cell Mol Physiol. 2016;310(6):L572–81. doi: 10.1152/ajplung.00417.2015 . 26747786 10.1152/ajplung.00417.2015 PMC4796258

[34] Zhang Y, Li X, Grailer JJ, Wang N, Wang M, Yao J, et al Melatonin alleviates acute lung injury through inhibiting the NLRP3 inflammasome. Journal of pineal research. 2016;60(4):405–14. Epub 2016/02/19. doi: 10.1111/jpi.12322 . 26888116 10.1111/jpi.12322

[35] Fukumoto J, Fukumoto I, Parthasarathy PT, Cox R, Huynh B, Ramanathan GK, et al NLRP3 deletion protects from hyperoxia-induced acute lung injury. Am J Physiol Cell Physiol. 2013;305(2):C182–9. doi: 10.1152/ajpcell.00086.2013 . 23636457 10.1152/ajpcell.00086.2013 PMC3725631

[36] Yu G, Zeng X, Wang H, Hou Q, Tan C, Xu Q. 14,15-epoxyeicosatrienoic Acid suppresses cigarette smoke extract-induced apoptosis in lung epithelial cells by inhibiting endoplasmic reticulum stress. Cellular physiology and biochemistry: international journal of experimental cellular physiology, biochemistry, and pharmacology. 2015;36(2):474–86. Epub 2015/05/15. doi: 10.1159/000430113 . 25968975 10.1159/000430113

[37] Abderrazak A, Syrovets T, Couchie D, El Hadri K, Friguet B, Simmet T, et al NLRP3 inflammasome: from a danger signal sensor to a regulatory node of oxidative stress and inflammatory diseases. Redox biology. 2015;4:296–307. Epub 2015/01/28. doi: 10.1016/j.redox.2015.01.008 25625584 10.1016/j.redox.2015.01.008 PMC4315937

[38] Hybertson BM, Lee YM, Cho HG, Cho OJ, Repine JE. Alveolar type II cell abnormalities and peroxide formation in lungs of rats given IL-1 intratracheally. Inflammation. 2000;24(4):289–303. Epub 2000/06/13. . 10850852 10.1023/a:1007092529261

