# Supplementary figures and images for: Vitexin attenuates lipopolysaccharide-induced acute lung injury by controlling the Nrf2 pathway
Source: PLoS One. 2018 Apr 25;13(4):e0196405. doi: 10.1371/journal.pone.0196405 (PMC5942793; doi:10.1371/journal.pone.0196405)

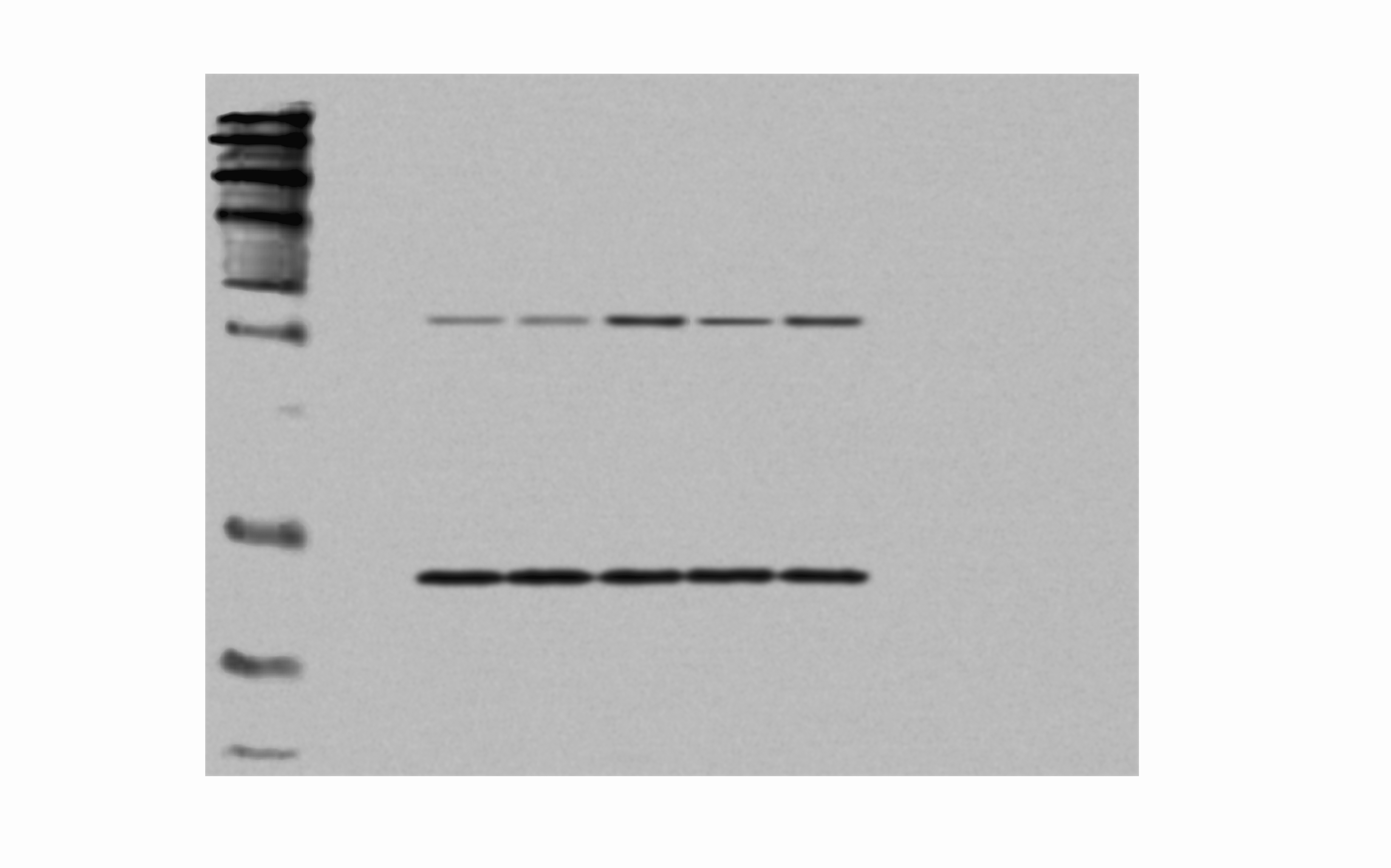

Supplement: S1 Fig — (TIF) [file pone.0196405.s001.tif]

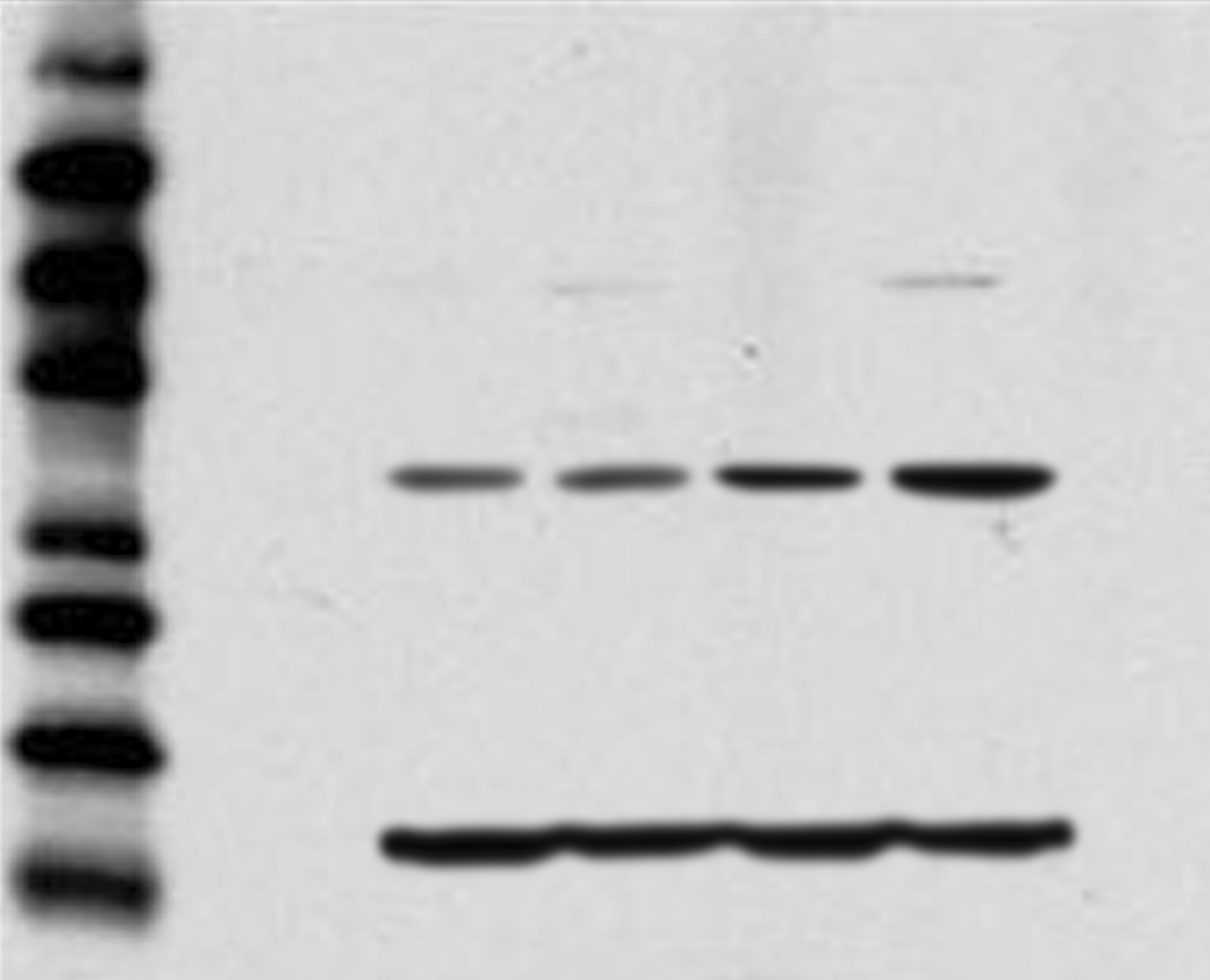

Supplement: S2 Fig — (TIF) [file pone.0196405.s002.tif]
